# Supplementary material for: Hybrid Models and Biological Model Reduction with PyDSTool
Source: PLoS Comput Biol. 2012 Aug 9;8(8):e1002628. doi: 10.1371/journal.pcbi.1002628 (PMC3415397; doi:10.1371/journal.pcbi.1002628)
Supplement: Text S4 — Complete source code for the PyDSTool package (version 0.88.120504). Includes API documentation and help files linking to web pages. This file is identical to the current public release on Sourceforge.net. (ZIP) [file pcbi.1002628.s004.zip › PyDSTool/html/PyDSTool.Points.PointInfo-class.html]

xml version="1.0" encoding="ascii"?


PyDSTool.Points.PointInfo


| Home | Trees | Indices | Help | PyDSTool |
| --- | --- | --- | --- | --- |

Package PyDSTool ::
Module Points ::
Class PointInfo

[hide private]

[frames] | no frames]

# Class PointInfo

source code

```
object --+
         |
        PointInfo
```

---

Structure for storing individual point labels and information
dictionaries within a Pointset object.

This class will not know the size of the Pointset it is associated
with, so index upper limits will not be checked in advance.

Do not use a PointInfo object as an iterator, as it is 'infinite' in
size! (It uses DefaultDicts as its internal storage, which return {} for
undefined labels.)


|  |  |
| --- | --- |
| Instance Methods | [hide private] |

|  |  |
| --- | --- |
| \_\_init\_\_(self, ptlabels=None)  x.\_\_init\_\_(...) initializes x; see x.\_\_class\_\_.\_\_doc\_\_ for signature | source code |

|  |  |
| --- | --- |
| mapIndices(self, ixMapDict) | source code |

|  |  |
| --- | --- |
| mapNames(self, themap)  Map labels, using a symbol map of class symbolMapClass. | source code |

|  |  |
| --- | --- |
| sortByIndex(self) | source code |

|  |  |
| --- | --- |
| sortByLabel(self) | source code |

|  |  |
| --- | --- |
| getIndices(self) | source code |

|  |  |
| --- | --- |
| getLabels(self) | source code |

|  |  |
| --- | --- |
| \_\_contains\_\_(self, key) | source code |

|  |  |
| --- | --- |
| \_\_getitem\_\_(self, key) | source code |

|  |  |
| --- | --- |
| \_\_setitem\_\_(self, key1, the\_rest) | source code |

|  |  |
| --- | --- |
| \_\_len\_\_(self) | source code |

|  |  |
| --- | --- |
| remove(self, key1, \*key2)  remove one or more items, keyed either by index or label. | source code |

|  |  |
| --- | --- |
| update(self, key1, key2=None, info=None) | source code |

|  |  |
| --- | --- |
| \_\_delitem\_\_(self, key) | source code |

|  |  |
| --- | --- |
| \_\_eq\_\_(self, other) | source code |

|  |  |
| --- | --- |
| \_\_ne\_\_(self, other) | source code |

|  |  |
| --- | --- |
| \_infostr(self, tab=0) | source code |

|  |  |
| --- | --- |
| \_\_repr\_\_(self)  str(x) | source code |

|  |  |
| --- | --- |
| \_\_str\_\_(self)  str(x) | source code |

**Inherited from `object`**:
`__delattr__`,
`__getattribute__`,
`__hash__`,
`__new__`,
`__reduce__`,
`__reduce_ex__`,
`__setattr__`


|  |  |
| --- | --- |
| Properties | [hide private] |

**Inherited from `object`**:
`__class__`


|  |  |
| --- | --- |
| Method Details | [hide private] |

|  |  |
| --- | --- |
| \_\_init\_\_(self, ptlabels=None)  *(Constructor)* | source code |

x.\_\_init\_\_(...) initializes x; see x.\_\_class\_\_.\_\_doc\_\_ for
signature

Overrides: object.\_\_init\_\_: *(inherited documentation)*

|  |  |
| --- | --- |
| \_\_repr\_\_(self)  *(Representation operator)* | source code |

str(x)

Overrides: object.\_\_repr\_\_: *(inherited documentation)*

|  |  |
| --- | --- |
| \_\_str\_\_(self)  *(Informal representation operator)* | source code |

str(x)

Overrides: object.\_\_str\_\_: *(inherited documentation)*

  


| Home | Trees | Indices | Help | PyDSTool |
| --- | --- | --- | --- | --- |

|  |  |
| --- | --- |
| Generated by Epydoc 3.0.1 on Fri May 4 15:24:07 2012 | http://epydoc.sourceforge.net |
